# Supplementary material for: MnO2-Ir Nanowires: Combining Ultrasmall Nanoparticle Sizes, O-Vacancies, and Low Noble-Metal Loading with Improved Activities towards the Oxygen Reduction Reaction
Source: Nanomaterials (Basel). 2022 Sep 1;12(17):3039. doi: 10.3390/nano12173039 (PMC9457901; doi:10.3390/nano12173039)
Supplement: Supplementary file 1 [file nanomaterials-12-03039-s001.zip › nanomaterials-1844603-supplementary.pdf]

MnO_2-Ir nanowires: Combining Ultrasmall Nanoparticle Sizes, O-vacancies and Low Noble-Metal Loading with Improved Activities Towards the Oxygen Reduction Reaction

Scarllett L. S. de Lima,[1] Fellipe S. Pereira,[2] Roberto B. de Lima,[2] Isabel C. de Freitas,[3] Julio Spadotto,[4] Jade Barreto,[5] Fernando Stavale,[5] Hector Aguilar Vitorino,[6] Auro A. Tanaka,[2] Marco A. S. Garcia,[2*] Anderson G. M. da Silva,[1*]

[1]Departamento de Engenharia Química e de Materiais-DEQM, Pontifícia Universidade Católica do Rio de Janeiro (PUC-Rio), Rua Marquês de São Vicente, 225 - Gávea 22453-900 Rio de Janeiro – RJ, Brazil.

[2]Departamento de Química, Centro de Ciências Exatas e Tecnologia, Universidade Federal do Maranhão (UFMA), São Luís - MA, Brazil.

[3]Departamento de Química Fundamental, Instituto de Química, Universidade de São Paulo, Av. Prof. Lineu Prestes, 748, 05508-000, São Paulo, SP, Brazil

[4]Materials Performance Centre, Department of Materials, University of Manchester, Manchester M13 9PL, UK

[5]Centro Brasileiro de Pesquisas Físicas, 22290-180, Rio de Janeiro, RJ, Brazil

[6]South American Center for Education and Research in Public Health, Universidad Norbert Wiener, Lima 15108, Peru.

**Corresponding author. E-mail: marco.suller@ufma.br, agms@puc-rio.br*

Table S1. Chemical and textural properties measured by ICP-OES and N_2-physisorption for the MnO_2 nanowires before and after the deposition of Ir NPs.

Catalyst	Ir Content (wt%)	Surface Area (m^2/g)	Pore Volume (cm^3/g)	Pore Diameter (Å)
MnO_2	-	123	0.42	6.1
MnO_2-Ir	1.2	130	0.41	6.1

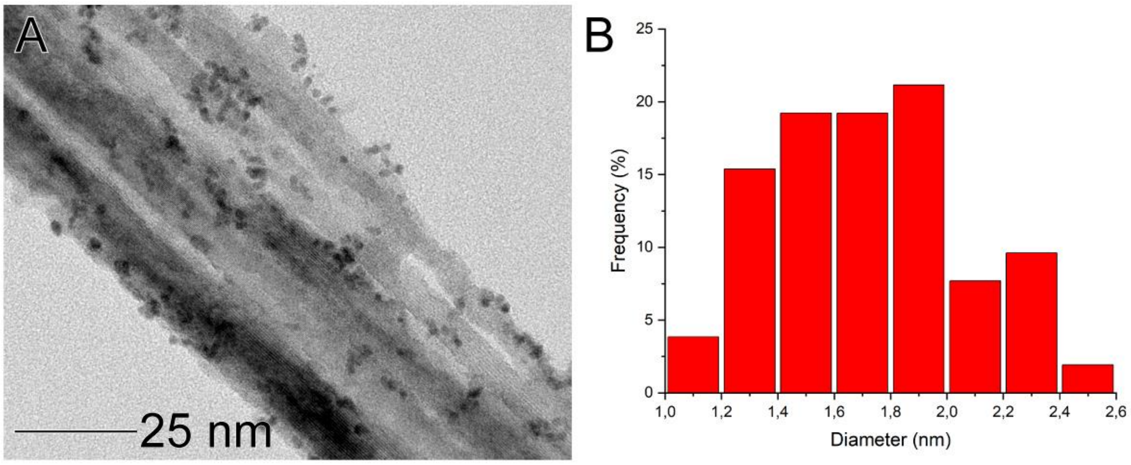

Figure S1. (A) HRTEM and (B) histogram of NPs distribution of MnO_2-Ir nanowires.

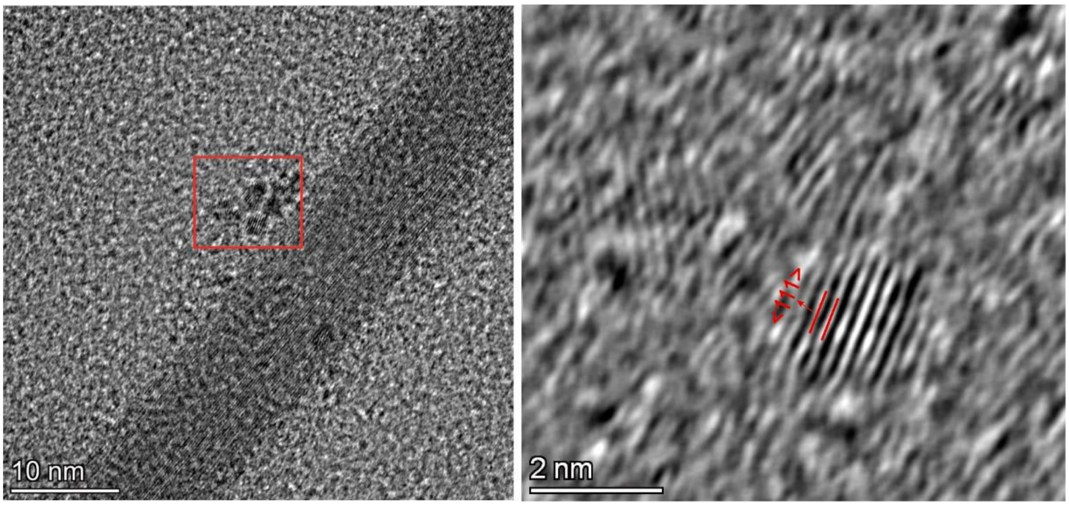

Figure S2. HRTEM Images of individual ultrasmall Ir NPs at the MnO_2 surface.

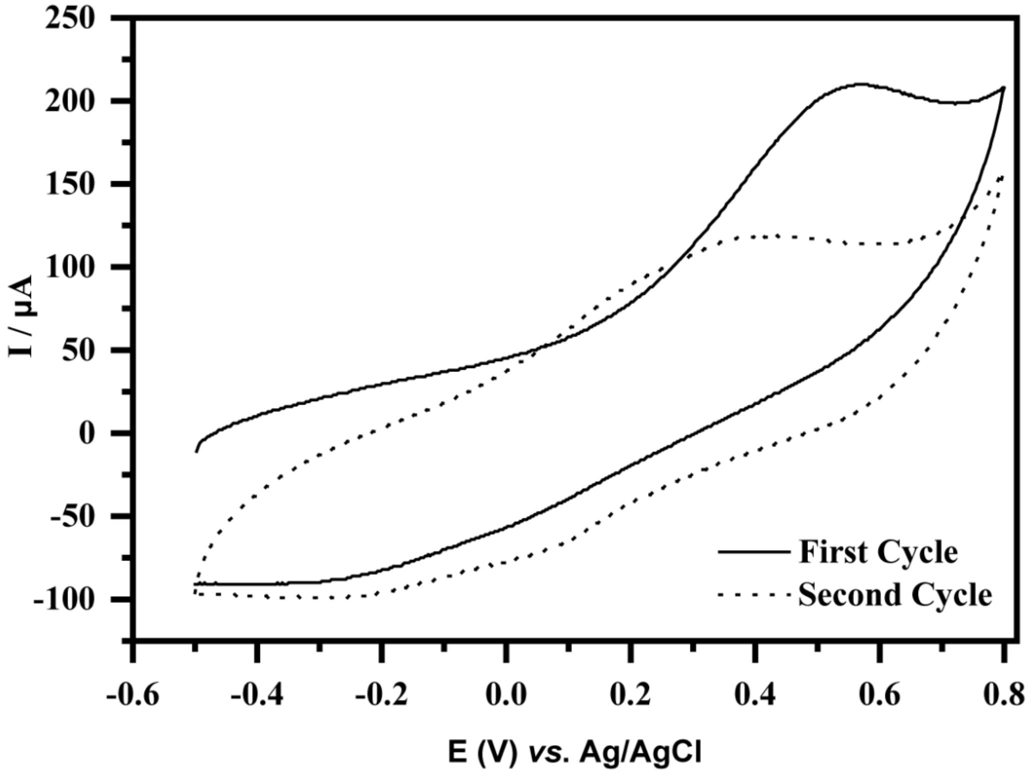

Figure S3. CO-stripping on MnO_2-Ir in 0.1 mol L^{-1} KOH solution with a potential scan rate of 25 mV s^{-1}.

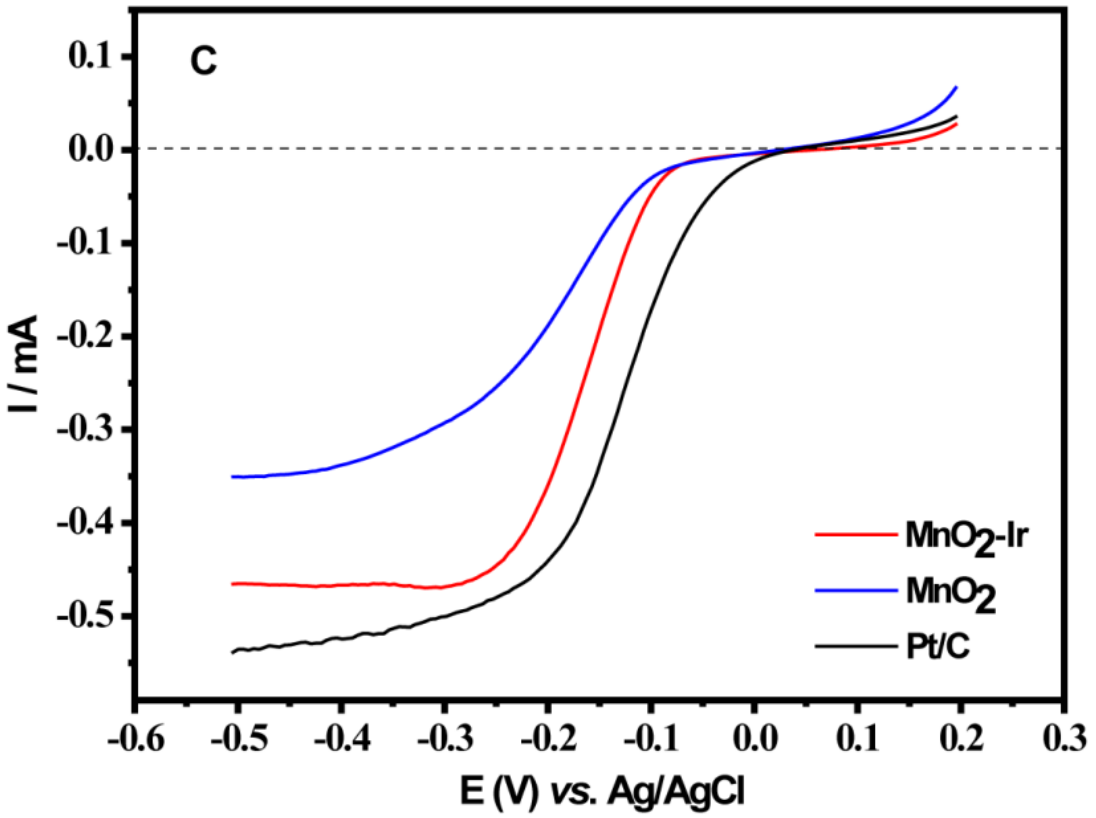

Figure S4. Polarization curves for the ORR on MnO_2, MnO_2-Ir, and 20.0 wt.% Pt/C materials, in 0.1 mol L^{-1} KOH solution, f= 1600 rpm, v = 5 mV s^{-1}, room temperature.

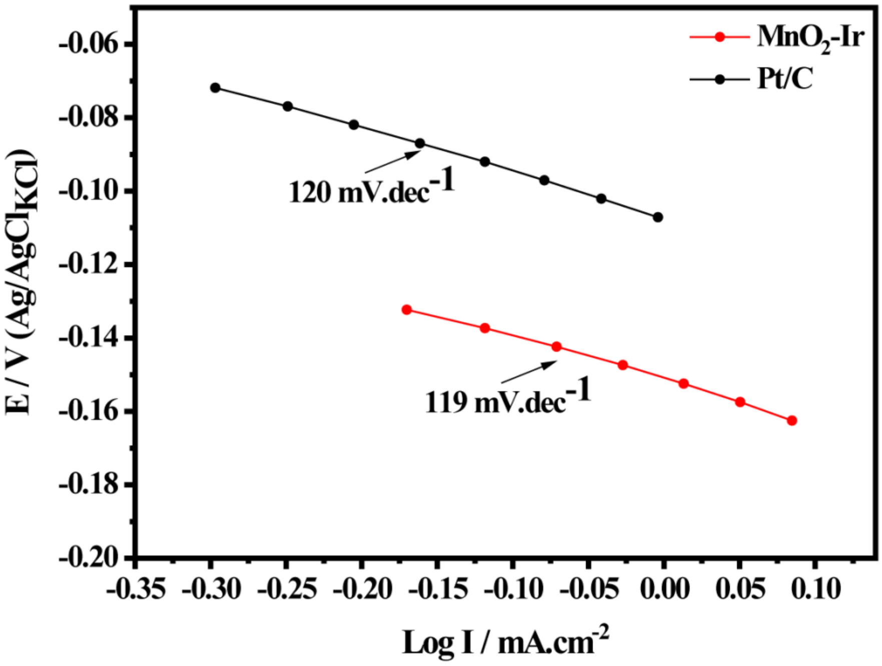

Figure S5. ORR Tafel plot for the Pt/C- and MnO_2-Ir electrocatalyst in O_2-saturated 0.1 mol L^{-1} KOH.

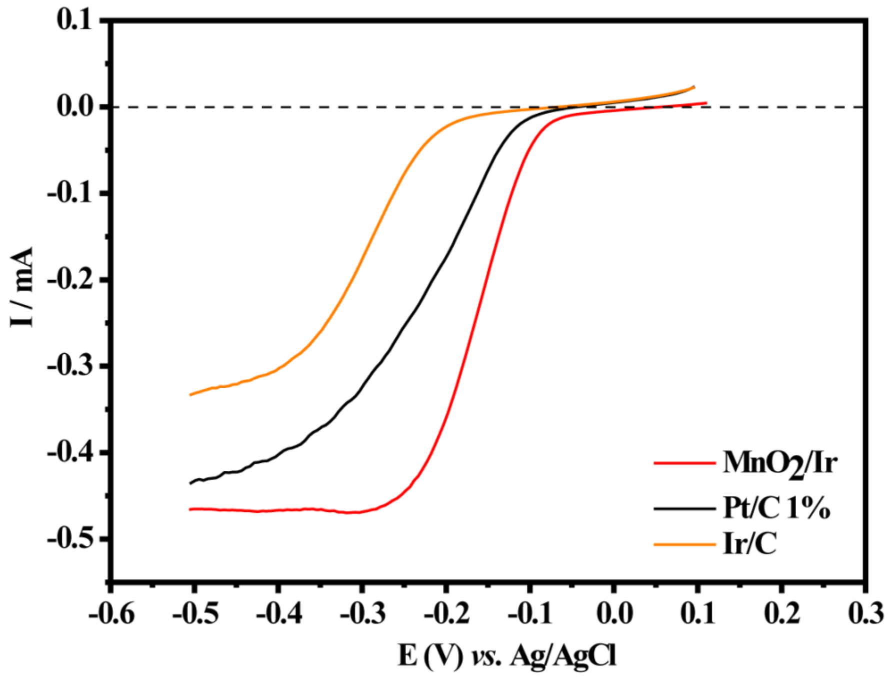

Figure S6. ORR polarization curves of the Ir-MnO_2, 1.2 wt% Pt/C, and Ir/C. Conditions: 0.1 mol.L^{-1} KOH, f = 1600 rpm, v = 5 mV.s^{-1} .
